# Organic animal farms increase farmland bird abundance in the Boreal region

**DOI:** 10.1371/journal.pone.0216009

**Published:** 2019-05-15

**Authors:** Andrea Santangeli, Aleksi Lehikoinen, Tanja Lindholm, Irina Herzon

**Affiliations:** 1 The Helsinki Lab of Ornithology, Finnish Museum of Natural History, University of Helsinki, Helsinki, Finland; 2 Helsinki Institute of Sustainability Science, HELSUS, University of Helsinki, Helsinki, Finland; 3 Department of Agricultural Sciences, University of Helsinki, Helsinki, Finland; Wageningen Universiteit, NETHERLANDS

## Abstract

Agriculture is a primary driver of biodiversity loss worldwide, and several expensive schemes have been designed to make modern farming landscapes more hospitable for wildlife. One such market-based mechanisms is the agri-environment-climate schemes (AES) in the European Union (EU). AES compensate farmers for reducing land-use intensity and maintaining or introducing biodiversity-rich habitats. Despite their high costs, impacts of AES vary by measure, region and taxonomic group considered, and have rarely been studied over large areas covering an entire country. Here we assess the country-wide impact of several AES measures on bird abundance using citizen science data on birds and detailed information on AES take up from across Finland. We report a positive impact of organic animal farming on abundance of all farmland associated birds. This effect was particularly strong for insectivorous species, species that are associated to farmyards and long-distance species. None of the other AES measures considered for study did show any relationship with bird abundance. Overall, these findings highlight the potential positive impact that some compensatory measures, such as organic animal farming, may have on wildlife. Traditional animal husbandry is based on grazing of animals and restriction on external inputs, similarly to what is stipulated under organic production contract. As such, traditional animal husbandry may represent an effective landscape management tool for restoring or maintaining threatened species and ecosystems in rural areas of the EU. Ultimately, the apparent lack of a measurable effect of the other AES considered here supports the current move towards evidence-based regional targeting of compensatory measures, so as to concentrate scarce resources to where they can yield the highest ecological benefits.

## Introduction

Agriculture is one of the most important drivers of biodiversity loss globally [1]. While the expansion of agriculture causes loss of native ecosystems, the intensification of land-use within existing agricultural landscapes often causes declines of species adapted to traditional man-managed agro-pastoral systems [2, 3]. In Europe, agriculture represents a dominant land-use type and supports considerable levels of biodiversity [4]. Across the continent, farmland-associated taxa are showing severe declines owing to increasingly intensive production regimes heavily based on use of chemical inputs, as well as simplification of the landscape and growing mechanization [3–5].

To counter the detrimental impacts of farmland intensification on biodiversity in the European Union (EU), a policy mechanism nowadays officially called agri-environment-climate schemes (hereafter AES) compensates farmers for the costs they bear when reducing land-use intensity and maintaining or introducing biodiversity-rich habitats. AES were introduced in 1992 as an obligatory policy element to all EU member states [6]. AES offer farmers a diversity of measures with a potential to benefit farmland wildlife, including birds: e.g. diversified crop rotation, introduction of buffer and biodiversity strips, and environmental fallows, each with specific environmental objectives. Farmers may also opt for organic production payment, which used to be a measure under AES until 2013, and currently represents a separate measure independent of AES. It is the only support measure targeted to the whole farm system. Adoption of AES by farmers is voluntary and currently about 25% of the agricultural area in the EU is under some form of AES or organic production contracts [7]. Overall, AES budgets are substantial, amounting to nearly €20 billion for the whole period 2007–2013 [7]. This often exceeds the budgets available for other means of nature conservation, such as establishing and managing protected areas [6].

While the spending for AES is conspicuous, the ecological effectiveness of these measures appears broadly positive but highly variable [6, 8]. General increases in farmland biodiversity following AES uptake have been observed, but effectiveness of AES largely varies according to the structure and management of the surrounding areas, and the recent history of the region [6]. Many AES measures broadly target wildlife communities, such as farmland birds, rather than single taxa, and while implemented locally, the main objective of this policy tool is to enhance the status of farmland wildlife at the landscape level. There is a large body of literature addressing the ecological effectiveness of AES measures [6]. However, most studies are restricted to one or few specific AES measures, to one or few species, or are limited in space. In order to increase our understanding of the relative effectiveness of AES measures and identify those that yield the most positive impacts on farmland wildlife, there is a need for studies of wide taxonomic and spatiotemporal coverage and of multiple AES measures implemented at the same time [9].

Moreover, there is a strong geographical bias in the studies assessing AES effectiveness towards intensively cultivated landscapes in central and western Europe [10]. In these structurally simple landscapes where up to 20% of semi-natural habitats remain, impacts of AES may be highest [10]. Conversely, impacts of AES may be lowest in complex landscapes where more than 20% semi-natural habitats remain [10], such as the farmland-forest mosaic of northern Europe. In such landscapes, evidence for AES impacts is scarce and limited in space, species coverage or in the measures considered. In Finland, over 90% of agricultural land is under some AES or organic production measures [11]. Consequently, it is important to provide the evidence for the effectiveness of AES measures on biodiversity in each biogeographical region. Such evidence is paramount in supporting decision-making on prioritising specific schemes in each AES programming period, and ultimately improve the effectiveness of this expensive policy tool [12].

Here we assess the country-wide impact of several AES measures on birds using data collected by citizen scientists (i.e. amateur birdwatchers) from across Finland over a six years period. Specifically, we first assess the effect of several AES measures on the abundance of species associated with farmland while controlling for relevant landscape and land-use factors. Second, we explore whether the effect of specific AES measures on farmland birds differs based on species traits such as species main habitat, diet, migration ecology, and Red List status within the EU. Exploring the differential effect of AES measures on species with contrasting traits is relevant because species with different diet (e.g. insectivorous versus granivorous) and different reliance on farmland (e.g. species that breed and feed on farmland versus those that only feed on farmland) may be differently affected by various AES measures [13–15]. Similarly, from a broader species conservation perspective, it is also relevant to understand the impact of AES on species of different migration ecology and conservation status. This is because long-distance migrant species are declining across Europe [16, 17], and AES may at least partly help these, as well as other threatened species, on their breeding grounds with appropriate measures [14].

## Materials and methods

### Study system

**Fig 1:**
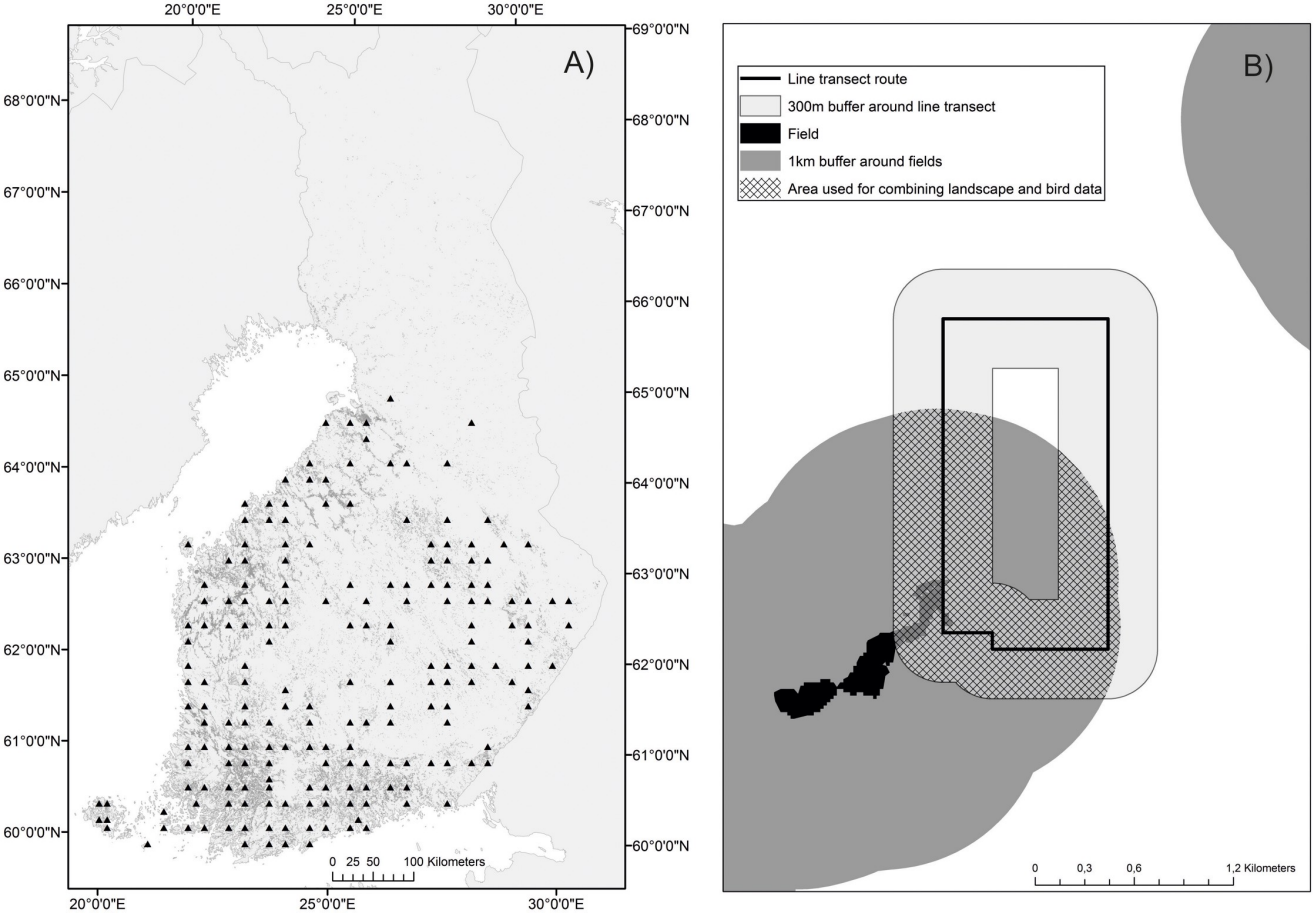
Study system and approach for combining data. A) The distribution of the line transects (black triangles) used for this study across the study region in South and Central Finland. B) An example showing how the landscape data where combined in space with the bird observation data from the line transects. The figure shows in dark grey the area within 1km from a field within which the landscape variables, centered at each farm, were interpolated. This area was in turn intersected with the 300m buffer zone (light gray area in panel A), and with the 1km buffer zone (not shown for simplicity) around a transect line (black continuous line). The average pixel value of the interpolated landscape variable (i.e. the relative influence of each land-use or Agri-environment scheme) within the intersection area (shown with hash pattern) was then extracted and related to the bird observations from that transect line.

The study took place across the entire terrestrial areas of Finland south of the Arctic Circle (Fig 1). In Finland, agricultural areas cover less than 10% of the territory. About 87% of all utilized agricultural areas are cultivated. The cultivated areas are largely (60% of the area) sown with grains such as spring barley and oat. Fodder grasslands cover 33% of the cultivated agricultural area, with pasture amounting to 4% of the total cultivated area [18]. Thus, contrary to most other Western European countries where farmland is dominant, in Finland most of the agricultural areas are typically surrounded by forests [19]. Although farmland represents only 7% of land cover in Finland, it supports a large wealth of biodiversity, including strongholds of several bird species of conservation concern in Europe, such as Lapwing *Vanellus vanellus* and Curlew *Numenius arquata* [20]. AES have been implemented in Finland since its accession to the EU in 1995 and have reached a wide coverage, with over 90% of Finnish farms having a payment contract for at least one AES or organic production [11].

### Bird survey data and species traits

We used six years of bird survey data collected during 2008–2013 through a standardized citizen science program which is part of a long term bird monitoring scheme running for over four decades in Finland [21]. We restricted the study to the above six years period due to the availability in the AES data and also because the bird surveys were standardized starting from 2006, see details below, and survey effort was rather constant after that. Bird surveys are based on a line transect method consisting of one early summer visit in which all birds are counted along a 6 km predetermined route [21]. The location of the transects is based on systematic sampling design, whereby transects are situated in 25 km interval across the whole country [22]. Surveys are carried out in the morning, typically between 03:00 and 10:00 h during June and under favourable conditions, i.e. no or very low wind or rain. All recorded observations are later converted into pairs, which then form the census unit (more details in [21]). These census units are then used to calculate the summed bird abundance per transect per year, see below.

Only the 46 farmland-associated species, as defined in [23], occurring in Finland were used for this study (see list in Table A in S1 File). From this pool of species, we excluded the Pheasant *Phasianus cholchicus*, as it is a farmed non-native species in Finland, and the Collared dove *Streptohelia decaocto* as it very rare and was never recorded in any of the transects used for this study. We obtained data on different ecological and life-history traits of the species from the literature. Specifically, birds were assigned to one of the four relevant habitat classes (hereafter *habitat*) following [23]: True farmland, farmland-forest edge (hereafter edge), forest, farmyard species (see Table A in S1 File for a list of all species considered for study and their traits). Main diet (hereafter *diet*) was also considered by using three classes: Granivorous, insectivorous, omnivorous species, following [24]. Migration ecology information (hereafter *migration ecology*) was obtained by [25], with three main categories: Partial migrant or sedentary, short-distance migrant, long-distance migrant (see Table A in S1 File). Finally, a variable representing the species Red List Status within the European Union (information obtained from: http://datazone.birdlife.org/info/euroredlist) was also considered (hereafter *status*). The species included in this study were classified as either Least Concern or Vulnerable (only these two threat statuses were associated to the studied species), hence the status variable was used as categorical variable with two classes.

### Agri-environment scheme and land-use data

We obtained AES and land-use variables from across the country from a database collated and managed by the Agency for Rural Affairs of Finland (www.mavi.fi). The database includes information on farm ownership, land-use, and agricultural and rural development support, among others. These data were provided as summed area of each of nine land-use and nine AES measures cover at the individual farm level (e.g. X hectares of spring cereal field area in farm Y; See Table 1 for a list of these variables and their description, as well as descriptive statistics reported in Table B in S1 File). We chose nine AES measures based on their plausible impact on birds (e.g. [26, 27]): environmental grassland, winter cover through light tillage, through stubble or through vegetation (three separate AES measures), biodiversity field, biodiversity and landscape management field, buffer zones, organic crop farm and organic animal farm (the latter two given as binary data at the level of a single farm being organic or not; details and definitions in Table 1). The measures are implemented on farms in various combinations and the maximum payment area for several measures varies among the country regions according to the targeting objectives. The land-use variables considered were winter and spring cereals, pasture, production grassland, hay meadows, non-cereal crops, green set-asides, non-field grassland and perennial grassland. Additional land-use variables available were the number of cattle, horse, pig and poultry per year per individual farm. During the study period, the organic production payment was a measure under the AES package and it could be implemented in combination with several other AES measures (such as buffer zones, biodiversity and landscape management field) but not with some measures commonly used in organic production (such as green manure grass). Because of this and the fact that it is a whole-farm measure, we consider it here as an AES measure.

**Table 1:** List of the 22 variables depicting the land-uses (LU) and agri-environment scheme (AES) measures on farm considered for this study along with their description and original unit of measure.

Variable name	Group	Description	Unit
Cattle	LU	N. of cattle	Count
Horse	LU	N. of horses	Count
Pig	LU	N. of pigs	Count
Poultry	LU	N. of poultry	Count
Winter cereal	LU	Total area covered by winter cereals	Hectares
Spring cereal	LU	Total area covered by spring cereals	Hectares
Production grassland	LU	Total area covered by production grasslands (mainly for animal fodder)	Hectares
Pasture	LU	Total area covered by pasture	Hectares
Hay meadow	LU	Total area covered by hay meadow	Hectares
Non-cereal crop	LU	Total area covered by crops other than cereals	Hectares
Green setaside	LU	Total area covered by several types of non-production grassland fields (fallows of various ages)	Hectares
Perennial grassland	LU	Total area covered by grassland being non-tilled for at least 5 years	Hectares
Non-field grassland	LU	Total area of grass-dominated land outside of fields (mostly uncultivated semi-natural meadows and pastures), can be subsidised under Biodiversity and landscape management	Hectares
Environmental grassland	AES	Total area covered by grassland on peat soils, grassland on fields within underground aquafer areas or grassland fallows[28]	Hectares
Winter cover (light tillage)	AES	Total area covered by fields under light tillage or direct seeding	Hectares
Winter cover (stubble)	AES	Total area covered by fields under stubble	Hectares
Winter cover (vegetation)	AES	Total area covered by grasslands of several types that stay over winter	Hectares
Biodiversity field	AES	Total area covered by [28]green manure grassland	Hectares
Biodiversity and landscape management	AES	Total area covered by land under management for biodiversity (includes semi-natural grasslands, biologically valuable forest ecotones, field margins and forest islands, fields important for endangered species)	Hectares
Buffer zone	AES	Total area covered by buffer zones: a field parcel under grass, along main ditches or waterways; on average of 15 m wide	Hectares
Organic crop farm	AES	A crop farm being either conventional or certified organic or in transition period (in Finland, includes also animal farms that certify only fields but not animals)	Binary
Organic animal farm	AES	An animal farm being either conventional or certified organic or in transition period (both animals and fields are certified)	Binary

### Combining bird survey and landscape data

All land-uses and AES were provided at the resolution of the individual farm. Therefore, we used spatial interpolation based on the inverse distance weight (hereafter IDW) to produce a continuous surface representing the effect of each of the landscape variables around the farm (see e.g. [29] for a similar approach). The effect of each variable was assumed to be highest towards the center of the farm and progressively decreasing up to zero at 4 km distance from the farm center. This distance was set because in Finland most fields belonging to a farm locate within 2 km from the farm center, while the effect of these fields may spill over to the immediate surroundings, progressively decreasing with the distance to the farm center. After the interpolation surface was produced across the whole landscape, we applied a mask that considered only the area within 1 km of each field (a spatial layer depicting all fields, irrespective of their specific use, was obtained from CORINE land cover maps 2012 and a 1 km buffer around them was created; Fig 1B). The remaining area (e.g. forests, lakes, mires) was excluded. This step resulted in interpolated maps representing the relative influence value for each of the 22 landscape variables from across the study region and for each of the study years separately from within 1km distance of each field. Next, we created a buffer of 300 m and 1 km around each bird line transect. We then used zonal statistics tools in ArcGIS to calculate the average relative influence value from across the pixels within 300 m and 1 km around each bird line transect per year for each of the 22 interpolated landscape variables (Fig 1B). High relative influence values indicate a high coverage of each land-use or AES surrounding the line transect, or a high concentration of organic animal or crop farms. This allowed to match the summed bird abundance at each line transect in each year with the average interpolated value of each landscape variable in that year within two spatial scales around the line transect. These two spatial scales were chosen as they are deemed to represent the immediate landscape around the transect line, within 300m, where birds have been observed, as well as the landscape available within a larger surrounding area that could still affect bird abundances along the transect line, e.g. through spillover effects [30].

In combining the landscape and the bird survey data, we also considered that some AES measures are ecologically relevant for the winter season, and as such they have been linked to bird counts performed in the following summer. Thus, AES measures such as winter cover through light tillage, stubble or vegetation, were matched with bird observations recorded in the following breeding season.

Finally, we derived in GIS two variables used as proxies for broad landscape structure and land-use intensity. As a proxy for landscape structure we considered the percentage of area covered by fields (hereafter *field area*) within a 5km radius around each line transect. As a proxy for land-use intensity we calculated the average field parcel size (in hectares; hereafter *parcel size*) of all fields occurring within the same 5km radius as above. This radius distance was chosen in order to represent large scale landscape processes while retaining relevance to the area surrounding the survey locations, as compared to considering a much larger radius.

### Statistical analyses

Because the focal habitat of this study is farmland, we excluded all line transects above the latitude of 65° North from the dataset, as farmland is extremely scarce there (Fig 1). For similar reasons, we also excluded line transects whereby the field cover within a 300m buffer radius around the transect line was less than five hectares. While the latter threshold is somewhat arbitrary, it was chosen because below that value the area of farmland is so scarce that is deemed to support negligible abundances of farmland associated birds. This filtering resulted in a total of 190 individual line transects scattered across the country, each surveyed on average 2.5 years between 2008 and 2013. The overall sample size for analyses (i.e. the overall number of surveys per line transect per year) was 468 individual units. Prior to analyses, we log transformed and then scaled all predictor variables, and transformed the line transect identity and the year when the survey was performed into categorical variables.

Next we assessed the level of collinearity between all the 22 landscape variables (see Table 1), plus the two additional variables of parcel size and field area using variance inflation analyses (VIF). We did this VIF analysis by considering landscape variables at the 300m and at the 1km scale. These analyses revealed that cattle was highly correlated (r > 0.8) with production grassland (silage) at both 300m and 1km scales. Hence, we excluded cattle from the following analyses to avoid multicollinearity. All other variables were very weakly correlated with each other (i.e. all had VIF < 4; [31], indicating that they can be included in the same model. We then built a set of linear mixed models (with Poisson distribution and log link function) which included in each case the summed bird abundance per line transect per year as the response, and the identity of the line transect as a random factor. The latter aimed to account for potential pseudoreplication due to repeated surveys of the same line transect over multiple years within the study period. Moreover, in each model parcel size and field area cover within 5 km were included as fixed continuous covariates to account for landscape scale patterns, and the year as a fixed categorical covariate to account for between year variation in bird abundance. This model structure represents the backbone structure of the linear mixed models. Because moderate overdispersion (dispersion parameter between 1.5 and 3) was apparent, we used a quasi-Poisson correction of the standard errors [31] implemented with the function glmmPQL in R software [32] for all models. This approach was recommended in such a situation under minimal overdispersion [31]. Because the quasi-Poisson is not a real distribution, the output does not include any information criterion or likelihood ratio value. We thus here show the results from the full model. This is appropriate in this case because the variables included in the models have been selected based on a specific ecological rationale.

The main focus of this study is in the effect of AES measures on bird abundances, whereas relevant land-use variables were only aimed to be controlled for as they may explain part of the variation in bird abundance. Therefore, we first added the uncorrelated set of land-use variables (n = 12) to the backbone model and inspected the results. From this model, we identified the land-use variables that were significant (p < 0.05; Table C in S1 File). We next built a model based on the backbone structure but with the addition of the significant land-use variables. To this model we added all the AES variables (n = 9) and reported the full results of this model, interpreting the results based on the p values and effect sizes of the AES variables. We used this approach for testing the effect of AES measures on summed bird abundance separately at the 300m and 1 km scales (i.e. repeating analyses using the AES measures and land-use variables calculated within 300m and 1km radius around the line transects). We also tested for non-linear (i.e. quadratic) effects of AES variables for which the linear effects were significant, but none of the non-linear effects were significant (p > 0.1) and were omitted from the results for clarity.

Finally, we test whether the effect of specific AES measures on farmland birds differs based on species traits. This was done by considering interactions between the significant AES variables (e.g. those that according to the above models significantly affected bird abundance) and each species trait. We tested the interaction with each trait by running four separate models, one per each trait. Each of these models had the same basic structure as detailed above but it also included the specific trait and its interaction with the relevant AES variable. For these models, the sample unit was the summed abundance of birds of each trait per transect per year.

All models detailed above we run separately for the two spatial scales considered (one based predictor variables extracted within the 300m buffer, and one based on the 1km buffer) as they are correlated, i.e. one includes the other. This resulted in one model testing the impact of AES variables on bird abundance, and four separate models, one for each interaction between AES measures and each of the four trait variables. These five models were run at the 300m and 1km scale, overall resulting in ten separate models.

Models were validated by inspecting the residuals [31], but no clear patterns in the residual distribution plots were observed that would indicate a violation in the model assumptions. Furthermore, we also checked for potential spatial autocorrelation in the residuals of the final models using spatial correlograms [31], but none of these showed any signs of spatial autocorrelation.

## Results

**Fig 2:**
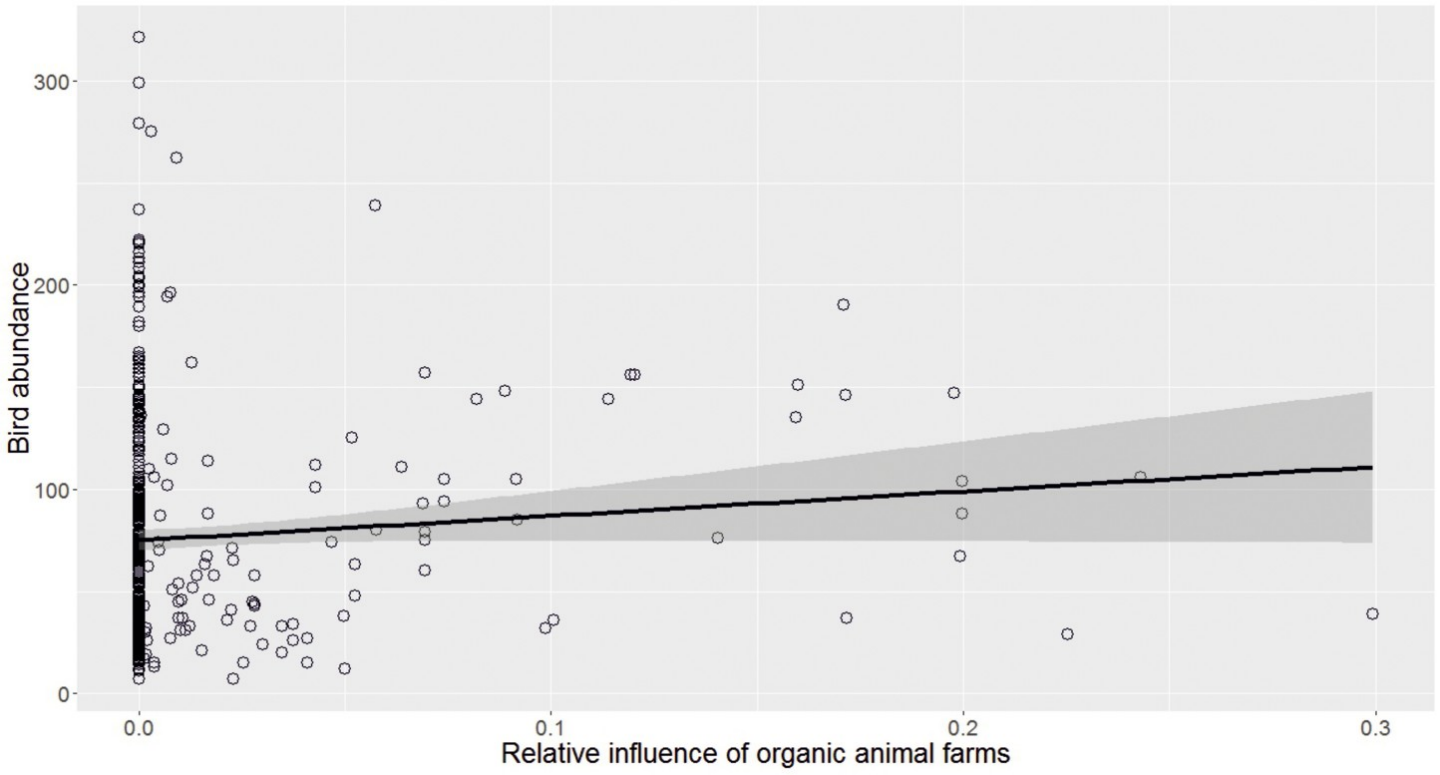
Effects of organic animal farms on overall bird abundance. The positive relationship between the relative influence of organic animal farms (averaged within a 300m radius from each transect line) and summed bird abundance across South and Central Finland between 2008 and 2013 based on the original untransformed data.

Overall, bird abundance was positively correlated with three variables: the percentage cover of fields within 5 km from the transect line, the relative influence of non-field grassland (i.e., semi-natural pastures and meadows) and presence of organic animal farms (both already certified as organic or in transition; Table 2 and Fig 2). The above results were consistent at both the 300m and 1 km scale. Of the above, only organic animal farms are subsidised under the agri-environmental programme. None of the other AES variables considered had a significant effect on bird abundance at any of the two spatial scales.

**Table 2:** Results of the generalized linear mixed model relating summed farmland associated bird abundance (response) with nine agri-environment scheme (AES) variables (rows highlighted in grey shade) while controlling for relevant land-use and other landscape variables as well as year. Results refer to models based at the 300m and the 1km scale (see Methods and Table 1 for more details on the model and the variables used).

Variable	300 m scale				1 km scale			
	β	SE	t	p	β	SE	t	p
Intercept	2.29	16.54	0.14	0.890	-0.29	16.80	-0.02	0.986
Field area	0.03	0.01	5.30	< 0.001	0.03	0.01	5.17	< 0.001
Parcel size	-0.06	0.08	-0.78	0.435	-0.07	0.08	-0.86	0.389
Year	0.00	0.01	0.09	0.924	0.00	0.01	0.25	0.804
Perennial grassland	-0.03	0.02	-1.30	0.195	-0.03	0.02	-1.54	0.125
**Non-field grassland**	**0.07**	**0.03**	**2.19**	**0.029**	**0.07**	**0.03**	**2.10**	**0.037**
Environmental grassland	0.02	0.03	0.73	0.464	0.02	0.03	0.77	0.443
Winter cover (light tillage)	0.00	0.04	0.04	0.966	0.01	0.04	0.33	0.743
Winter cover (stubble)	0.06	0.04	1.46	0.145	0.08	0.04	1.75	0.082
Winter cover (vegetation)	-0.04	0.05	-0.87	0.385	-0.04	0.05	-0.84	0.399
Biodiversity field	-0.01	0.02	-0.38	0.702	0.00	0.02	-0.08	0.933
Biodiversity and landscape management	-0.02	0.03	-0.54	0.591	-0.03	0.03	-1.00	0.319
Buffer zone	-0.01	0.02	-0.51	0.611	-0.01	0.02	-0.27	0.791
Organic crop farm	0.02	0.03	0.54	0.589	0.02	0.03	0.54	0.591
**Organic animal farm**	**0.04**	**0.02**	**2.37**	**0.018**	**0.05**	**0.02**	**2.34**	**0.020**

**Fig 3:**
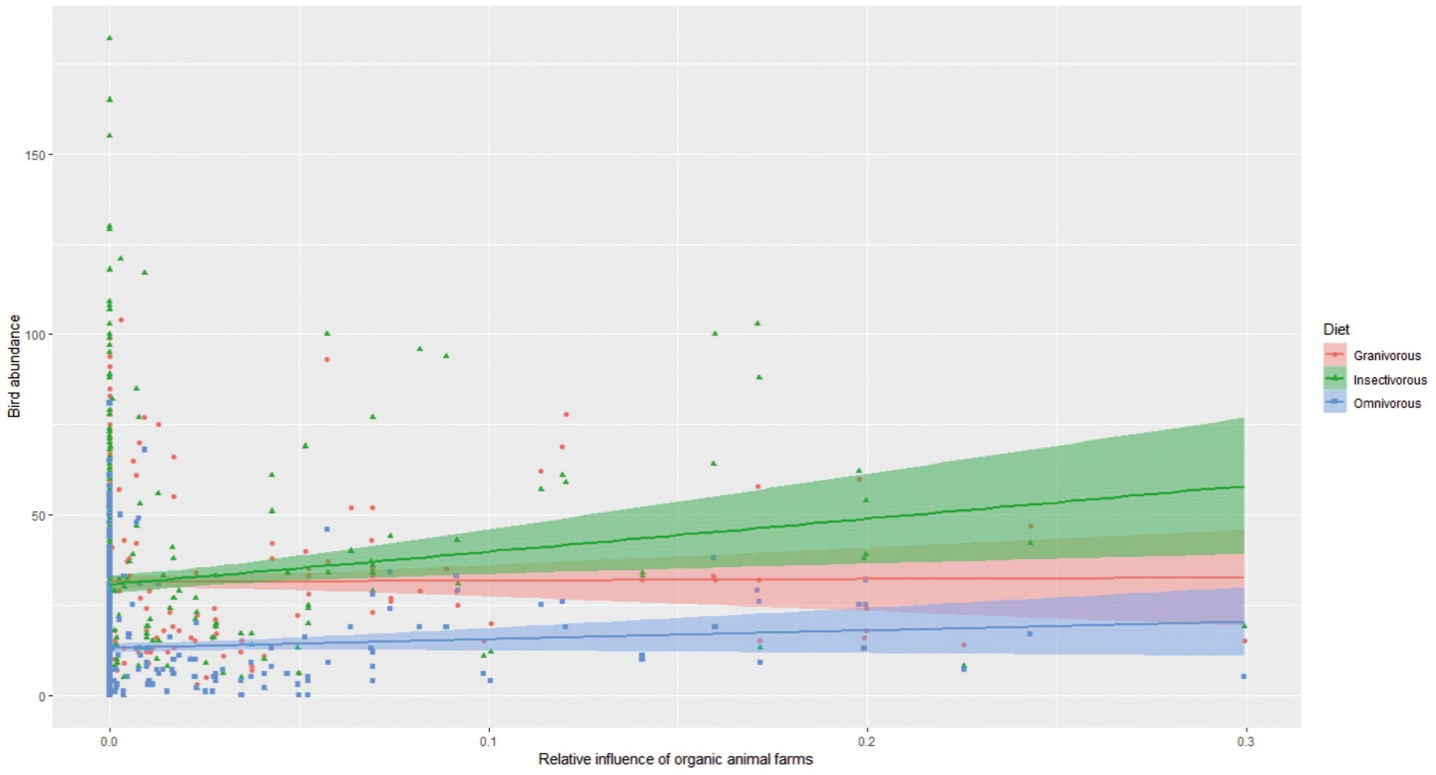
Effects of organic animal farms on bird abundance by diet class. The differential effect of relative influence of organic animal farms (averaged within a 300m radius from each transect line, see Fig 1b) on the summed abundance of granivorous, insectivorous and omnivorous (including raptors) birds. Values are based on the original untransformed data. The results of the interaction between relative influence of organic animal farms and birds diet are presented in Table C in S1 File.

**Fig 4:**
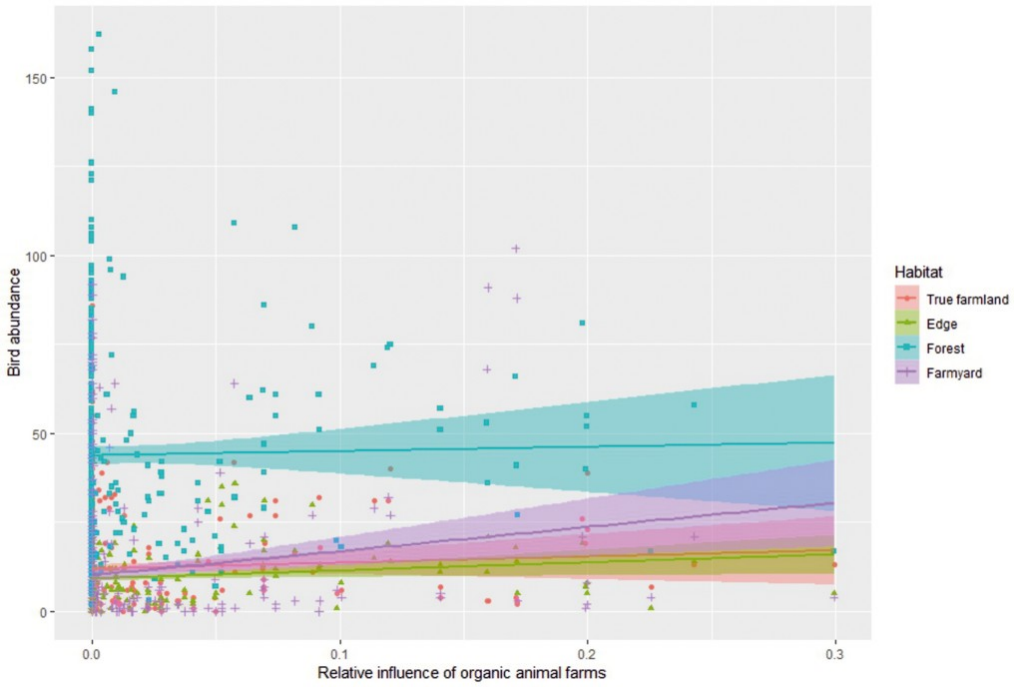
Effects of organic animal farms on bird abundance by habitat class. The differential effect of relative influence of organic animal farms (averaged within a 300m radius from each transect line, see Fig 1b) on the summed abundance of birds separated by their main habitat (true farmland, farmyard, edge and forest species). Values are based on the original untransformed data. The results of the interaction between relative influence of organic animal farms and birds habitat are presented in Table D in S1 File.

**Fig 5:**
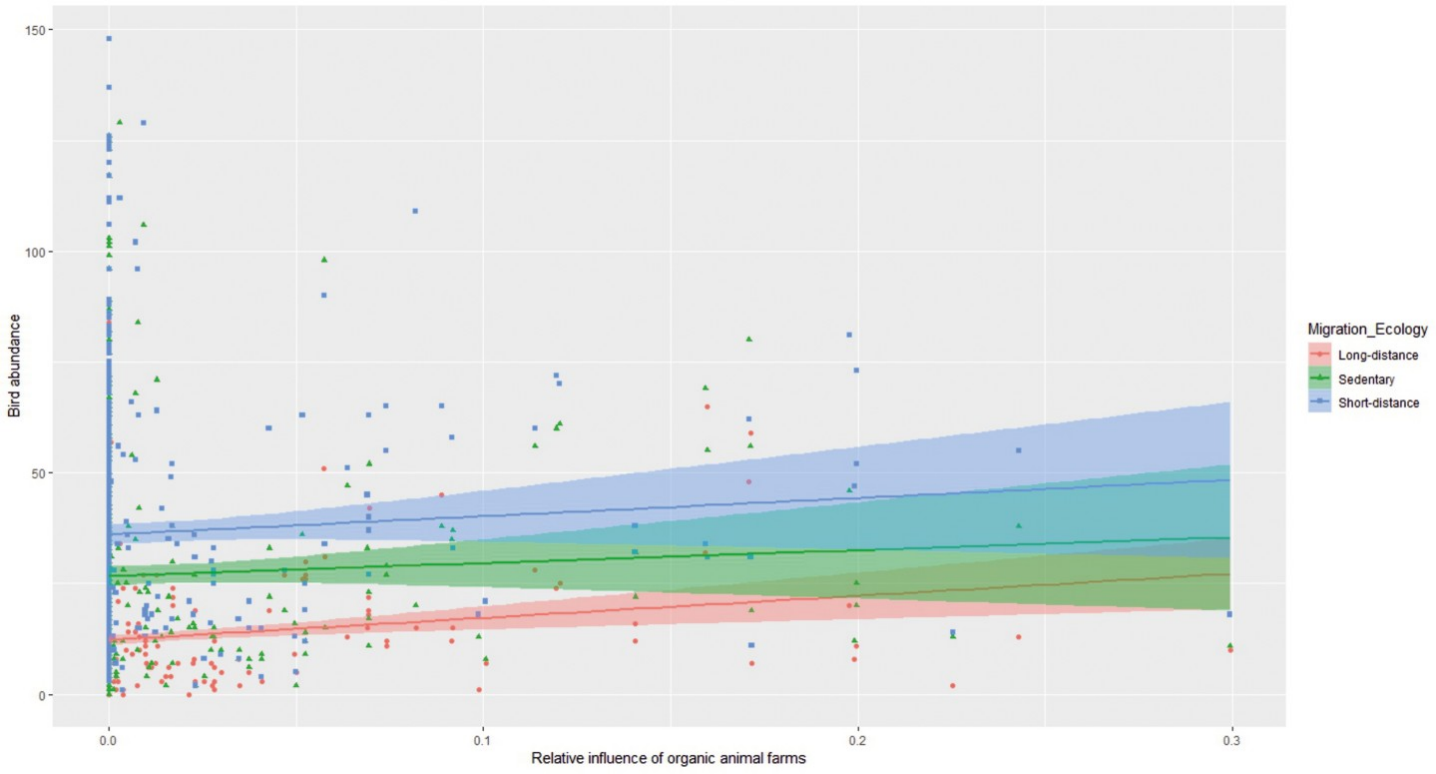
Effects of organic animal farms on bird abundance by migration ecology class. The differential effect of relative influence of organic animal farms (averaged within a 300m radius from each transect line, see Fig 1b) on the summed abundance of birds separated by their main migration ecology (long-distance migrant, short-distance migrant, sedentary, which includes also partial migrant species). Values are based on the original untransformed data. The results of the interaction between relative influence of organic animal farms and birds migration ecology are presented in Table E in S1 File.

Next, we quantified the interactive effects of organic animal farming on birds abundance with four species-trait variables (i.e. diet, habitat, migration ecology and Red List status). These interactions revealed that the effect of organic animal farms on bird abundance was significantly more positive for insectivorous species compared to granivorous ones at both spatial scales, and more positive for omnivorous species compared to granivorous ones at the one-km scale (Fig 3; Table D in S1 File). The effect of organic animal farms on bird abundance was also mediated by the main habitat used by the species. Specifically, the effect was significantly more positive for farmyard species compared to true farmland, edge or forest species at both spatial scales, and more positive for edge species compared to forest ones at the scale of 1 km (Fig 4; Table E in S1 File). With regards to the species migration ecology, we found that the effect of organic animal farms was significantly more positive for long-distance migrant species compared to short-distance or sedentary (including partial migrant) species (Fig 5; Table F in S1 File). Finally, the effect of organic animal farms on bird abundance was unaffected by the species status being Vulnerable or Least Concern (Table G in S1 File).

## Discussion

Here we assessed the effect of several measures under agri-environment schemes (AES), including organic farming, on the abundance of farmland- associated birds across Finland. We show that, among all AES measures considered, organic animal farming was the only measure that had a measurable positive effect on bird abundance. Specifically, we found that the effect of organic animal farms was most positive on insectivorous, and to a lower extent, on omnivorous birds, as well as on farmyard or long-distance migrant species. With regard to land-use types, we found that non-field grassland (i.e. semi-natural pastures and meadows) had a measurable positive effect on bird abundance. The above results were consistent at both 300m and 1km spatial scales considered. None of the other AES measures, including organic crop farm contract, had any significant effect on bird abundance.

The organic production is the only full-farm system support scheme that stipulates a coherent package of several management obligations. On top of these, farmers with organic production contracts may chose some additional AES fitting their situations, but so that the maximum payment ceiling per farm is observed. In Finland, most of organic farms raise cattle and sheep [33]. The animals raised at organic animal farms are kept on pastures during the vegetative season, and for a longer time period as compared to those raised on conventional farms [33]. Animals raised by organic standards in Finland are typically left grazing on pastures from May to September. This period largely overlaps with the breeding season of the farmland bird species considered in this study. Also in winter the animals have access to outdoors. The animal feed must contain at least 60% of roughage (e.g., grass, hay), of which at least 80% should come from the farm itself (i.e., the farm has to have sufficient field area to return manure [33]). Finally, use of antibiotics is restricted [33], which in turn may boost invertebrate numbers, a critical resource for farmland birds [34]. Such farm management provides birds with several critical resources. Presence of animals and their dung on pastures and use of manure have been proven to boost abundance of aerial [35] as well as soil invertebrates [36], which represent a key food source for most farmland associated bird species, particularly during the breeding period [4, 34, 37]. This enriched food availability may largely explain our result that the effect of organic animal farms is most positive for insectivorous species and farmyard species. The latter are typically species that breed near or at the farmyard (e.g. *Hirundo rustica*, *Delichon urbicum*, *Sturnus vulgaris*, *Passer montanus*) and forage in the nearby farmland fields [38]. These species may more readily take advantage of the boosted invertebrate abundance at organic animal farms. By consuming large amounts of insects, many of which are considered pest species, insectivorous birds provide valuable ecosystem services [39]. Thus, the result we found has implications not only for wildlife conservation on farmland, but may also have spill over implications on the associated ecosystem services.

Previous research done in Finland reported a positive effect of organic farms (including both animal and crop only farms) on a small selection of strictly farmland birds (those that breed and forage on open farmland; [15]). We show here that the effect of organic animal farms on bird abundance is twice more positive than that of organic crop farms, highlighting the importance that livestock animals, mainly cattle, have on birds when they are raised according to organic standards [37]. While evidence that the positive effects of organic farming remain local (field level) for several taxa [40], we show here that a positive effect is also apparent at a level beyond the specific field parcel. This conclusion is supported by the positive effect of organic animal farming on bird abundance at both 300m and 1km scales.

Notably, all other AES measures considered showed negligible effect on bird abundance across the country. Recent reviews of the impact of conservation measures, including AES, on farmland biodiversity in Europe suggest that these are largely mediated by landscape context, land-use intensity and the ecological contrast that selected measures create with the rest of the farmland landscape [9, 10]. Boreal farmland landscapes are composed of mosaics of small-scale open farmland alternating with non-field grassland and other biotopes, mainly forests. Therefore, in this heterogeneous landscape, the contrast created by the AES measures tested in this study may not have been large enough to trigger a measurable positive effect on bird abundance, with the exception of the organic animal farms discussed above. This pattern was highlighted by other similar studies in Finland and beyond [13, 15]. Moreover, about 90% of Finnish farms adopt at least one AES measure but usually a combination of several measures. Thus, in Finland AES may already cover a large enough area, thereby potentially further reducing the contrast created by a single AES measure. This may in turn prevent detecting any measurable impact of specific AES measures on birds, as also suggested by [9].

Previous studies provided evidence for benefits of extensive grasslands and especially fallows on farmland birds [41] at the landscape level in Southern Finland. However, in our country-level analysis, extensive grassland and fallows appeared to have no effect on birds abundance. This discrepancy may relate to such fields being much commoner in other regions of Finland compared to the most-productive South of the country where most other studies took place [42]. However, we found a positive effect of only non-field grassland on bird abundance. These areas are semi-natural pastures and meadows of traditional types. So far, only a weak association between this land-use and birds has been shown [43]. If sufficiently large, they may provide important nesting and food resources for farmland birds, thereby explaining our result. This type of habitat is also among the most threatened habitats in Finland according to a new report [44]. The fact that presence of such habitats as land-use type rather than their management status under AES was more effective on birds may indicate an insufficient coverage of these areas, and particularly of importance to birds, by management contracts. Yet these non-field grasslands of semi-natural kind are a backbone of High Nature Value farming in Europe [45]. Ensuring the preservation of this habitat will likely aid in farmland bird conservation as well as conservation of a wealth of other taxa associated with extensive semi-natural pastures. Conversely, the broadly positive effect of field cover in the landscape on bird abundance was expected given the set of species considered. A similar finding was also reported in a study conducted in southern Finland [46].

We acknowledge that the study has some limitations that need to be born in mind when interpreting the results. These limitations largely relate to the resolution of the data used and to the fact that this study was designed *a posteriori*, when all data where already collected. The land-use and AES data where only available to us as aggregated information at the farm level. This may potentially cause some bias in the land-use and most of the AES variables due to the fact that these are variables are all centered at the center of the farm. However, our approach to use an interpolation method, and countrywide data, should minimise any error due to the specific location of each land-use or AES parcel as compared to an alternative approach based on calculating the distance, rather than the interpolated density, of the farm center to the bird survey location. This low resolution may partly explain the lack of an effect found for most AES and land-use variables considered. However, we consider the above issue of less relevance with regards to the organic crop and animal farm variables as these have relevance across the whole farm area rather than to specific field parcels. Therefore, the interpolation approach used to derive these two variables is deemed to be the most conservative.

We provide evidence, albeit correlative, that organic animal farming is positively associated with the abundance of farmland associated birds, particularly insect-eating species. Conversely, the fact that most other AES measures considered in this study have no measurable effect on bird abundance calls for a careful evaluation and use of this expensive tool. Failure to do so may result ineffective use of scarce conservation resources [47, 48]. Evaluating and reconsidering such expensive scheme may be even more crucial where the additionality of AES may be limited (e.g. support to grasslands in regions with already high cover of grasslands; [9]). In these contexts, supporting the current move towards evidence-based regional targeting may yield better ecological outcomes for the money spent.

## Supporting information

S1 File **Tables A-G Table A**. **List of the 44 farmland associated species and their traits**. Habitat categories include 1 = true farmland, 2 = farmland-forest edge, 3 = forest, 4 = farmyard species. Diet includes I = insectivorous, G = granivorous and terrestrial herbivores, O = omnivorous and birds of prey. Migration includes PN = partial migrant or sedentary, S = short-distance migrant, L = long-distance migrant. Red List includes the status of the species as relevant to the European Union, LC = Least concern, VU = Vulnerable. **Table B**. **Descriptive statistics of the variables used for this study.** Descriptive statistics (mean and standard deviation) of the 22 variables depicting the land uses (LU) and agri-environment scheme (AES) measures considered for this study (see Table 1 in main manuscript for a description of each variable).The statistics refer to the interpolated variables relative to the area within 300m and 1km around each line transect used for this study (more details in the methods section of the main manuscript).**Table C. Results of the model to identify important land-use variables explaining bird abundance.** Results of the model aimed at identifying the most important (here considered to be when p < 0.05) land use variables explaining bird abundance at the 300m and 1km scale. Only the significant land use variables (here highlighted in bold font) from this model were controlled for in the following models (Table 2) that aimed at quantifying the effect of agri-environment scheme measures on bird abundance while controlling for relevant the land use variables (i.e. perennial grassland and non-field grassland). **Table D.** Results of the model testing for the interactive effect of organic animal farm density and birds diet (3 classes: insectivorous, granivorous and omnivorous) on the abundance of farmland associated bird species in Finland. **Table E. Results of model on interaction between organic animal farms and birds diet.** Results of the model testing for the interactive effect of organic animal farm density and birds main habitat (4 classes: True farmland, edge, forest, farmyard species) on the abundance of farmland associated bird species in Finland. **Table F. Results of model on interaction between organic animal farms and birds migration ecology.** Results of the model testing for the interactive effect of organic animal farm density and birds migration ecology (3 classes: Sedentary (which also includes partial migrants), short-distance migrant, long-distance migrant) on the abundance of farmland associated bird species in Finland. **Table G. Results of model on interaction between organic animal farms and birds Red List status.** Results of the model testing for the interactive effect of organic animal farm density and birds Red List status in the European Union (2 classes: Vulnerable vs Least Concern) on the abundance of farmland associated bird species in Finland.
(PDF)
