# Supplementary material for: Organic animal farms increase farmland bird abundance in the Boreal region
Source: PLoS One. 2019 May 15;14(5):e0216009. doi: 10.1371/journal.pone.0216009 (PMC6519808; doi:10.1371/journal.pone.0216009)
Supplement: S1 File — Tables A-G Table A. List of the 44 farmland associated species and their traits. Habitat categories include 1 = true farmland, 2 = farmland-forest edge, 3 = forest, 4 = farmyard species. Diet includes I = insectivorous, G = granivorous and terrestrial herbivores, O = omnivorous and birds of prey. Migration includes PN = partial migrant or sedentary, S = short-distance migrant, L = long-distance migrant. Red List includes the status of the species as relevant to the European Union, LC = Least concern, VU = Vulnerable. Table B. Descriptive statistics of the variables used for this study. Descriptive statistics (mean and standard deviation) of the 22 variables depicting the land uses (LU) and agri-environment scheme (AES) measures considered for this study (see Table 1 in main manuscript for a description of each variable).The statistics refer to the interpolated variables relative to the area within 300m and 1km around each line transect used for this study (more details in the methods section of the main manuscript).Table C. Results of the model to identify important land-use variables explaining bird abundance. Results of the model aimed at identifying the most important (here considered to be when p < 0.05) land use variables explaining bird abundance at the 300m and 1km scale. Only the significant land use variables (here highlighted in bold font) from this model were controlled for in the following models (Table 2) that aimed at quantifying the effect of agri-environment scheme measures on bird abundance while controlling for relevant the land use variables (i.e. perennial grassland and non-field grassland). Table D. Results of the model testing for the interactive effect of organic animal farm density and birds diet (3 classes: insectivorous, granivorous and omnivorous) on the abundance of farmland associated bird species in Finland. Table E. Results of model on interaction between organic animal farms and birds diet. Results of the model testing for the interac [file pone.0216009.s001.pdf]

Table A.

Species latin name	Habitat	Diet	Migration	Red List EU
Acrocephalus dumetorum	2	I	L	LC
Acrocephalus palustris	2	I	L	LC
Acrocephalus schoenobaenus	2	I	L	LC
Alauda arvensis	1	G	S	LC
Anthus pratensis	1	I	S	VU
Apus apus	4	I	L	LC
Asio otus	3	O	S	LC
Buteo buteo	3	O	S	LC
Carduelis cannabina	4	G	S	LC
Carduelis carduelis	4	G	PN	LC
Carduelis chloris	3	G	PN	LC
Carpodacus erythrinus	2	G	L	VU
Charadrius dubius	1	I	L	LC
Columba livia	4	O	PN	LC
Columba oenas	3	G	S	LC
Columba palumbus	3	G	S	LC
Corvus frugilegus	4	O	S	LC
Corvus monedula	3	O	PN	LC
Corvus corone cornix	3	O	PN	LC
Crex crex	1	I	L	LC
Delichon urbica	4	I	L	LC
Emberiza citrinella	3	G	PN	LC
Emberiza hortulana	1	G	L	LC
Emberiza schoeniclus	2	G	S	LC

Falco tinnunculus	3	O	S	LC
Gallinago gallinago	1	I	S	LC
Hirundo rustica	4	I	L	LC
Lanius collurio	2	I	L	LC
Locustella fluviatilis	2	I	L	VU
Locustella naevia	2	I	L	LC
Motacilla alba	4	I	S	LC
Motacilla flava	1	I	L	LC
Numenius arquata	1	O	S	VU
Oenanthe oenanthe	4	I	L	LC
Passer domesticus	4	G	PN	LC
Passer montanus	4	I	PN	LC
Perdix perdix	1	G	PN	LC
Pica pica	3	O	PN	LC
Saxicola rubetra	2	I	L	LC
Sturnus vulgaris	4	I	S	LC
Sylvia communis	2	I	L	LC
Tringa totanus	1	I	S	VU
Turdus pilaris	3	I	S	VU
Vanellus vanellus	1	I	S	VU

Table B.

	300 m scale		1 km scale	
**Variable name**	**Mean**	**SD**	**Mean**	**SD**
Cattle	15.89	18.41	15.99	17.35
Horse	0.72	2.07	0.65	1.63
Pig	11.80	42.29	12.75	37.35
Poultry	187.09	1094.22	165.40	868.71
Winter cereal	0.87	1.91	0.88	1.80
Spring cereal	19.39	13.15	19.58	12.82
Production grassland	10.66	7.80	10.70	7.53
Pasture	1.42	1.64	1.41	1.54
Hay meadow	0.15	0.68	0.15	0.59
Non-cereal crop	2.33	3.31	2.35	3.12
Green setaside	1.18	1.35	1.19	1.26
Perennial grassland	2.65	2.51	2.66	2.36
Non-field grassland	1.08	4.63	1.11	4.75
Environmental grassland	1.31	3.60	1.29	3.42
Winter cover (light tillage)	8.03	10.23	8.24	10.48
Winter cover (stubble)	5.48	10.10	5.54	9.67
Winter cover (vegetation)	8.78	12.90	9.00	12.74
Field biodiversity	0.93	2.05	0.97	2.01
Biodiversity measure	1.36	9.35	1.36	9.53
Buffer zone	0.14	0.50	0.16	0.54
Organic crop farm	0.07	0.13	0.07	0.12
Organic animal farm	0.01	0.04	0.01	0.04

Table C.

	300 m scale				1 km scale			
**Variable**	**Coeff**	**SE**	**t**	**p**	**Coeff**	**SE**	**t**	**p**
Intercept	-3.867	16.511	-0.23	0.815	-4.189	16.813	-0.25	0.804
Field area	0.023	0.005	4.36	< 0.001	0.025	0.005	4.71	< 0.001
Parcel size	-0.083	0.083	-1.01	0.314	-0.093	0.082	-1.14	0.256
Year	0.004	0.008	0.48	0.635	0.004	0.008	0.49	0.628
Horse	-0.014	0.025	-0.57	0.569	-0.008	0.025	-0.31	0.756
Pig	0.000	0.028	0.01	0.993	-0.023	0.030	-0.74	0.458
Poultry	0.019	0.021	0.89	0.372	0.024	0.020	1.15	0.251
Winter cereal	0.014	0.018	0.76	0.448	0.011	0.018	0.62	0.534
Spring cereal	0.075	0.040	1.88	0.062	0.064	0.036	1.74	0.082
Production grassland	0.018	0.031	0.57	0.568	0.025	0.032	0.79	0.431
Pasture	-0.047	0.025	-1.88	0.061	-0.040	0.025	-1.64	0.102
Hay meadow	-0.019	0.030	-0.64	0.526	-0.019	0.029	-0.64	0.523
Non-cereal crop	0.031	0.021	1.49	0.138	0.036	0.022	1.60	0.112
Green setaside	0.011	0.017	0.63	0.526	0.018	0.017	1.06	0.292
**Perennial grassland**	**-0.046**	**0.019**	**-2.37**	**0.019**	**-0.047**	**0.019**	**-2.46**	**0.014**
**Non-field grassland**	**0.084**	**0.033**	**2.56**	**0.011**	**0.093**	**0.032**	**2.92**	**0.004**

Table D.

Variable	300 m scale				1 km scale			
	**Coeff**	**SE**	**t**	**p**	**Coeff**	**SE**	**t**	**p**
Intercept	-0.171	18.371	-0.01	0.993	-2.536	18.519	-0.14	0.891
Field area	0.028	0.005	5.68	< 0.001	0.028	0.005	5.66	< 0.001
Parcel size	-0.061	0.078	-0.78	0.434	-0.061	0.078	-0.78	0.436
Year	0.002	0.009	0.17	0.864	0.003	0.009	0.30	0.766
Perennial grassland	-0.034	0.021	-1.62	0.106	-0.036	0.021	-1.74	0.082
Non-field grassland	0.091	0.035	2.63	0.009	0.085	0.034	2.50	0.013
Organic animal farm	-0.008	0.024	-0.32	0.746	-0.015	0.024	-0.64	0.520
Diet - insectivorous (ref: Granivorous)	0.009	0.024	0.38	0.700	0.008	0.024	0.35	0.727
Diet - omnivorous (ref: Granivorous)	-0.855	0.031	-27.62	< 0.001	-0.855	0.031	-27.68	< 0.001
**Organic * Diet - insectivorous (ref: Granivorous)**	**0.087**	**0.022**	**3.92**	**< 0.001**	**0.099**	**0.022**	**4.49**	**< 0.001**
**Organic * Diet - omnivorous (ref: Granivorous)**	**0.056**	**0.029**	**1.95**	**0.052**	**0.060**	**0.029**	**2.07**	**0.039**
Organic * Diet - insectivorous (ref: Omnivorous)	0.031	0.027	1.13	0.261	0.039	0.027	1.44	0.149

Table E.

Variable	300 m scale				1 km scale			
	**Coeff**	**SE**	**t**	**p**	**Coeff**	**SE**	**t**	**p**
Intercept	-1.283	21.934	-0.06	0.953	-3.409	22.121	-0.15	0.878
Field area	0.027	0.005	5.68	< 0.001	0.027	0.005	5.67	< 0.001
Parcel size	-0.058	0.077	-0.75	0.455	-0.058	0.077	-0.75	0.452
Year	0.002	0.011	0.15	0.880	0.003	0.011	0.25	0.806
Perennial grassland	-0.033	0.025	-1.34	0.179	-0.035	0.024	-1.42	0.155
Non-field grassland	0.104	0.037	2.82	0.005	0.099	0.036	2.71	0.007
Organic animal farm	0.033	0.036	0.93	0.352	0.035	0.035	0.99	0.321
Habitat - Edge (ref: True farmland)	-0.254	0.050	-5.10	< 0.001	-0.254	0.050	-5.11	< 0.001
Habitat - Forest (ref: True farmland)	1.288	0.037	34.58	< 0.001	1.288	0.037	34.69	< 0.001
Habitat - Farmyard (ref: True farmland)	-0.120	0.048	-2.49	0.013	-0.125	0.048	-2.59	0.010
Organic * Habitat - Edge (ref: True farmland)	0.033	0.044	0.74	0.462	0.029	0.044	0.66	0.508
Organic * Habitat - Forest (ref: True farmland)	-0.039	0.035	-1.12	0.264	-0.051	0.035	-1.47	0.140
**Organic * Habitat - Farmyard (ref: True farmland)**	**0.120**	**0.040**	**3.02**	**0.003**	**0.135**	**0.039**	**3.44**	**0.001**
**Organic * Habitat - Forest (ref: Edge)**	**-0.071**	**0.037**	**-1.95**	**0.051**	**-0.080**	**0.037**	**-2.19**	**0.029**
**Organic * Habitat - Farmyard (ref: Edge)**	**0.087**	**0.041**	**2.12**	**0.034**	**0.106**	**0.041**	**2.57**	**0.010**
**Organic * Habitat - Farmyard (ref: Forest)**	**0.158**	**0.031**	**5.15**	**< 0.001**	**0.186**	**0.031**	**6.06**	**< 0.001**

Table F.

Variable	300 m scale Coeff	SE	t	p	1 km scale Coeff	SE	t	p
Intercept	-1.035	17.873	-0.06	0.954	-3.412	18.029	-0.19	0.850
Field area	0.028	0.005	5.68	< 0.001	0.028	0.005	5.66	< 0.001
Parcel size	-0.062	0.078	-0.79	0.431	-0.061	0.078	-0.78	0.434
Year	0.002	0.009	0.17	0.862	0.003	0.009	0.30	0.761
Perennial grassland	-0.034	0.021	-1.67	0.096	-0.036	0.020	-1.79	0.073
Non-field grassland	0.089	0.034	2.60	0.010	0.083	0.034	2.46	0.014
Organic animal farm	0.106	0.026	4.04	< 0.001	0.113	0.026	4.30	< 0.001
Migration - Sedentary (ref: Long-dist. migrant)	0.749	0.031	23.95	< 0.001	0.750	0.031	24.01	< 0.001
Migration - Short-dist. migrant (ref: Long-dist. migrant)	1.051	0.030	35.12	< 0.001	1.053	0.030	35.18	< 0.001
**Organic * Migration - Sedentary (ref: Long-dist. migrant)**	**-0.080**	**0.026**	**-3.04**	**0.002**	**-0.091**	**0.027**	**-3.44**	**0.001**
**Organic * Migration - Short-dist. migrant (ref: Long-dist. migrant)**	**-0.079**	**0.025**	**-3.14**	**0.002**	**-0.089**	**0.025**	**-3.56**	**< 0.001**
Organic * Migration - Short-dist. migrant (ref:Sedentary)	0.002	0.022	0.08	0.933	0.002	0.022	0.09	0.932

Table G.

Variable	300 m scale Coeff	SE	t	p	1 km scale Coeff	SE	t	p
Intercept	0.343	21.808	0.02	0.987	-1.532	22.066	-0.07	0.945
Field area	0.027	0.005	5.66	< 0.001	0.027	0.005	5.65	< 0.001
Parcel size	-0.057	0.077	-0.74	0.461	-0.057	0.077	-0.74	0.462
Year	0.002	0.011	0.15	0.882	0.003	0.011	0.23	0.817
Perennial grassland	-0.033	0.025	-1.36	0.176	-0.034	0.024	-1.42	0.156
Non-field grassland	0.103	0.037	2.80	0.005	0.098	0.036	2.69	0.007
Organic animal farm	0.031	0.023	1.31	0.191	0.030	0.023	1.28	0.200
Red List Status - Vulnerable (ref: Least Concern)	-1.056	0.030	-35.40	< 0.001	-1.056	0.030	-35.37	< 0.001
Organic * Red List Status - Vulnerable (ref: Least Concern)	0.030	0.026	1.14	0.253	0.025	0.027	0.94	0.346
